# Influences on use of antibiotics without prescription by the public in low- and middle-income countries: a systematic review and synthesis of qualitative evidence

**DOI:** 10.1093/jacamr/dlae165

**Published:** 2024-10-25

**Authors:** Christie Cabral, Tingting Zhang, Isabel Oliver, Paul Little, Lucy Yardley, Helen Lambert

**Affiliations:** Centre for Academic Primary Care, Bristol Medical School, University of Bristol, 39 Whatley Road, Bristol BS8 2PS, UK; Centre for Academic Primary Care, Bristol Medical School, University of Bristol, 39 Whatley Road, Bristol BS8 2PS, UK; United Kingdom Health Security Agency, Chief Scientific Officer's Group, London, UK; Primary Care Research Centre, University of Southampton, Southampton SO16 5ST, UK; School of Psychological Science, University of Bristol, The Priory Road Complex, Priory Road, Clifton, Bristol BS8 1TU, UK; School of Psychology, University of Southampton, Southampton SO17 IBJ, UK; Department of Population Health Sciences, Bristol Medical School, University of Bristol, 39 Whatley Road, Bristol BS8 2PS, UK

## Abstract

**Objectives:**

Self-medication with antibiotics is common practice in many low- and middle-income countries (LMIC). This review synthesizes the qualitative evidence on influences on perceptions and practices in relation to self-medication by the public with antibiotics in LMIC.

**Methods:**

A systematic search was conducted of relevant medical, international and social science databases. Searching, screening, data extraction and quality appraisal followed standard methods. A meta-ethnographic approach was used for synthesis, starting with translation of studies and using a line-of-argument approach to develop the final themes.

**Results:**

The search identified 78 eligible studies. Antibiotics were understood as a powerful, potentially dangerous but effective medicine for treating infections. This perception was strongly influenced by the common experience of being prescribed antibiotics for infections, both individually and collectively. This contributed to an understanding of antibiotics as a rational treatment for infection symptoms that was sanctioned by medical authorities. Accessing antibiotics from medical professionals was often difficult logistically and financially. In contrast, antibiotics were readily available over the counter from local outlets. People viewed treating infection symptoms with antibiotics as rational practice, although they were concerned about the risks to the individual and only took them when they believed they were needed.

**Conclusions:**

A new model to explain self-medication with antibiotics is presented. This uses the socio-ecological model to integrate influences that operate at individual, community and wider socioeconomic levels, drawing on theories of medical authority and the medicalization and commercialization of health. Interventions to reduce overuse of antibiotics in LMIC need to address both clinical practice and community self-medication practices together.

## Introduction

Low- and middle-income countries (LMIC) have a high prevalence of communicable diseases, high rates of antibiotic use and face serious consequences from antibiotic resistance.^1–4^ Antimicrobial stewardship (AMS) in LMIC encounters challenges particular to these contexts, including underfunded healthcare systems and the related widespread availability of antibiotics without prescription from community retail outlets.^5–7^ Self-medication with antibiotics, usually obtained from retail outlets without prescription, is common practice in most LMIC.^5,8,9^ The difficulty of accessing formal healthcare (with qualified prescribers) in LMIC is a key driver of this practice,^10^ and in some countries health authorities may allow this practice to continue as a way to provide access to antibiotics.^11,12^ This leaves LMIC facing the ‘access-excess’ challenge: how to ensure access while restricting excess use.^13^ Understanding antibiotic use practices outside formal healthcare settings is particularly important for AMS in LMIC.

Consumption of unprescribed antibiotics is often framed as ‘irrational’ in the medical literature and attributed to deficits in knowledge and lower education levels.^14–17^ This ‘irrational’ use is attributed to a public knowledge deficit and is cited as a key barrier to AMS in LMIC community settings.^6,18^ However, ‘rational’ in this context refers solely to use that conforms to clinical guidelines and it has been argued that the labelling of antibiotics use as ‘irrational’ is part of a discourse that locates the problem in ‘others’ so that they can then be targeted for corrective intervention.^19^ Anthropological studies of local explanatory understandings of illness and treatment provide evidence of consumers’ own rationalities for antibiotic use.^20,21^ People draw on life-world and experiential knowledge from a range of sources including medical professionals, medicine dispensers and social norms to determine which symptoms to self-treat with antibiotics.^22,23^ In Asia, understandings of antibiotics combine traditional medicine paradigms and biomedical ideas into a knowledge hybrid that underpins both patient and professional practice, calling into question the common assumption that lack of knowledge is the problem.^24^ In Africa, socioeconomic factors and insufficient access to healthcare are recognized drivers of antibiotic use without prescription, although the association with low educational status remains the focus of many studies.^9^ Although the literature from South America is sparse, there is again a focus on local understandings, with an acknowledgement that the wider socioeconomic context also plays a role.^25–27^ Non-biomedical understandings influence antibiotics use in high-income countries (HICs) as well as LMIC, and shape perceptions of which symptoms require treatment, primarily because of clinical encounters within which clinicians link certain symptoms to the decision to prescribe antibiotics.^28,29^ Despite calls from social scientists to look beyond knowledge deficits when seeking to understand antibiotic use,^24,30^ the idea of ‘irrationality’ persists in the medical literature.^31–33^

The focus on knowledge, beliefs or even lay understandings ignores wider structural influences on antibiotic use.^21,24,30^ In the context of weak health systems and wider socioeconomic vulnerabilities, self-medication may be the only affordable or accessible treatment.^13,34^ In this way the underlying structural vulnerabilities in LMIC contribute to driving the use of antibiotics and the associated harms from AMR.^34^ Health inequalities caused by regulatory or other institutions of the state have been described as a form of structural violence,^35^ and regulatory controls that seek to restrict public access to antibiotics are likely to disproportionately impact the poorest.^36^ Wider societal structures are also connected to antibiotic use practices, both those that contribute to disease incidence (e.g. water and sanitation infrastructure) and socioeconomic pressures to work and produce,^21,30^ which are also felt most acutely among the poorest.

To our knowledge there has been no comprehensive synthesis of the qualitative evidence relating to self-medication with antibiotics in LMIC. There are some mixed-methods, biomedically framed reviews looking at predictors of self-medication that include small numbers of qualitative studies from LMIC,^10,37–39^ or specific LMIC regions.^9,40^ A recent systematic review^6^ of qualitative evidence examined the drivers of sale without prescription, focusing on the views of those working in community drug retail outlets. This review identified four key influences: (i) the business orientation and need to make profits; (ii) customer expectations; (iii) lack of regulation or enforcement; and (iv) staff lack of knowledge. This review aims to provide a complementary synthesis of the qualitative evidence pertaining to the views, experiences and practices of the public. The aim of this review is to synthesize the evidence regarding perceptions and practices of people living in LMIC that influence their use of antibiotics without prescription, using an anthropological lens to examine the findings.

## Methods

This review used standard Cochrane methods^41^ for searching for relevant studies, screening and data extraction. Then a meta-ethnographic approach^42^ was used for the synthesis of extracted data. A study protocol is provided as Supplementary material (available as Supplementary data at *JAC-AMR* Online). We followed Preferred Reporting Items for Systematic Reviews and Meta-Analyses (PRISMA) guidelines.

### Specifying the question

The SPICE question-framing tool was used to specify the question:

*Setting:* LMICs.

*Perspective:* non-medically trained people, including the general public, patients and caregivers.

*Phenomenon of interest:* anything that influences the use of antibiotics without prescription including beliefs about antibiotics and antibiotic resistance, cultural norms and practices, financial influences at individual and societal level, health delivery systems, regulations and enforcement.

*Comparison:* none.

*Evaluation:* qualitative or mixed-methods study types that use methods including ethnographic, semi-structured or in-depth interviews, or focus group discussions.

### Exclusion criteria

1. Studies not reporting data from LMIC settings.
2. Studies reporting views of those who have received medical training including biomedical doctors and medical students, nurses, pharmacists or other biomedical health professionals.
3. Studies reporting views of those employed in clinical settings including hospitals, private clinics offering biomedical treatment and village clinics, and retail pharmacies or drug (medicine) shops.
4. Studies not reporting data on phenomena of interest.
5. Studies about non-human antibiotic use.
6. Studies not reporting qualitative data.

### Literature search

We searched three large health science databases: MEDLINE, Embase and CINHAL. We also searched specialist databases for LMIC literature: WHOLIS (World Health Organization Library Database), GIM (Global Index Medicus), LILACS (Latin American and Caribbean Health Sciences Literature) and Anthropology Plus. Search strategies were tailored for each database and consisted of: [Terms for antibiotics] AND [Terms for self-medication] AND [Terms for qualitative study types] (see detailed search strategy in Supplementary material). No date and language limits were set. Searches were first run in March 2022 and updated in November 2023. Separate searches were run in each database and then the search results were combined and de-duplicated in EndNote X9 before screening all results for records relevant to review. Citation tracking was carried out for studies cited in any reviews identified by the search to check for additional studies.

### Study selection

Screening was conducted in two phases. In the first phase, titles and abstracts of a sample of 400 records (around 10%) were independently carried out by two researchers (T.Z. and C.C.) for eligibility. Researchers discussed their inclusion decisions until consensus could be researched, and inclusion criteria clarified. One researcher (T.Z.) then screened all remaining records for inclusion. Full-text versions of the remaining studies were obtained. In the second phase, around 25% (*n* = 60) of full-text records were screened independently by the same two researchers (T.Z. and C.C.) against inclusion and exclusion criteria and any disagreements were resolved by discussion. T.Z. then screened all remaining full-text records. Studies published in any other language were translated into English using Google translation for the purpose of determining whether they met inclusion criteria.

### Data extraction

Data extraction was carried out by T.Z. using a pre-agreed data extraction form covering study characteristics (author, publication year, country, aim, study design, methods, data collection period, setting, population) and study findings that related to the phenomena of interest, including both themes and corresponding primary data reported in the included studies.

### Critical appraisal

The quality assessment was conducted at the same time as data extraction. Two researchers (T.Z. and C.C.) together evaluated the quality of 10% of records using Popay’s quality appraisal tool for qualitative studies,^43^ and T.Z. evaluated the remaining records. Studies were grouped into higher-, medium- and lower-quality studies according to whether they were judged as good quality in all seven quality criteria (high), four to six quality criteria (medium), or less than four quality criteria (low). Quality criteria were not used to exclude studies from the review, but to inform the order in which studies were incorporated into the data synthesis, which started with the higher-quality studies.

### Synthesis

The findings were synthesized using a meta-ethnography approach.^42,44^ This is an inductive and interpretive approach to synthesis, which involves comparing the constructs or themes from the included studies to identify those with similar or related meanings in a process that is similar to the constant comparative method that is used in grounded theory.^42^ Constructs from different studies provided interpretations of the same phenomena (or aspects thereof) from different perspectives and were combined to produce a new interpretation of the evidence as a whole. Starting with the oldest high-quality study, the extracted themes from each study were put into a matrix, keeping themes that capture similar meanings from different studies together in the same or adjacent rows. Higher-quality studies were added in order of publication, then medium-quality studies and finally low-quality studies were added to the matrix. Drawing on both theme descriptions and supporting primary data, study themes were ‘translated’ into one another, so that those describing different aspects of the same phenomena could be combined and higher-order constructs generated. A translation or summary description was written at the beginning of each row that captured the common meaning and preserved the study authors’ conceptual interpretations.^45^ Translation was an iterative process, with an early summary translation developed once themes from a few primary studies could be combined into a higher-order construct and then revisions made each time another study had new relevant themes and data. As the final studies were added, fewer changes were needed to the overall summaries, indicating that most aspects of the phenomena had been captured. A line-of-argument approach was then used to combine the translated constructs into the final synthesis.

## Results

**Figure 1.**
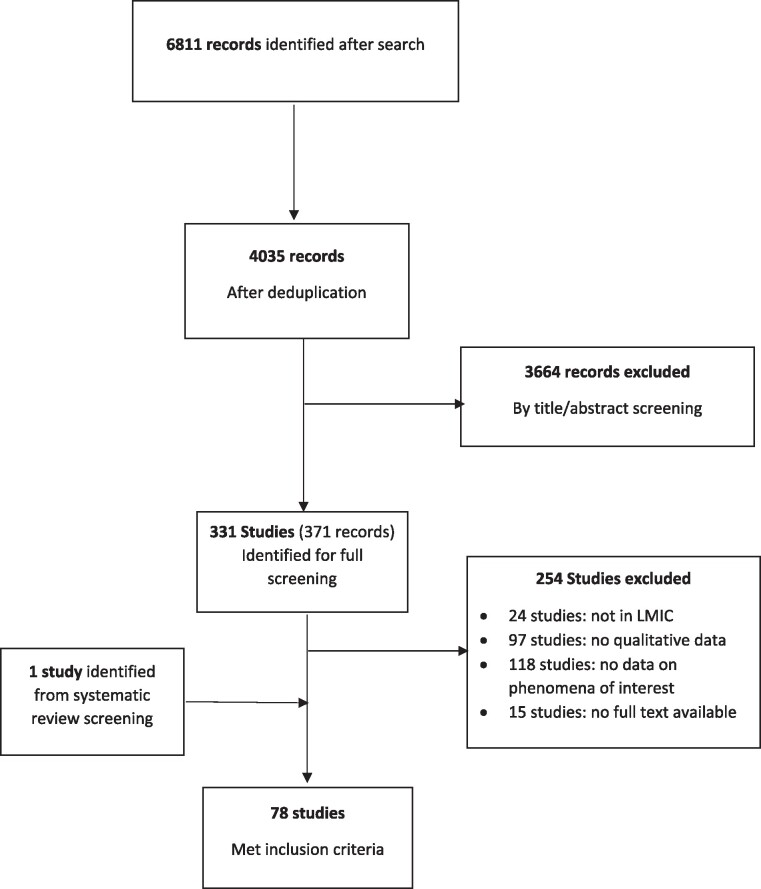
Study identification flow chart.

The searches identified 6811 records, of which 2776 were excluded after deduplication and 3664 were then excluded on screening of title and abstract. The remaining 371 records reported on 331 studies, for which full texts were obtained and screened. This identified 78 studies that met the inclusion criteria, including 1 study identified from screening systematic reviews identified by this search (see Figure 1).

### Study characteristics

The 78 included studies were all published between 1996 and 2023, with just over 60% published since 2019. Fifty-five studies used qualitative design and the other 23 used mixed methods. There were 71 studies focused on a single LMIC, 6 on multiple LMIC, and 1 on a mix of HICs and LMIC. The total number of participants from all the included studies is difficult to determine because not all studies specified their sample size (see Table 1 for individual study population descriptions); the total number of participants from the included studies that did specify sample size was 3360. Using the WHO regions to consider the geographical distribution of the studies: there were 26 studies from 19 countries in the African Region (*n* > 1395), 22 studies from 6 countries in the South-Eastern Region (*n* > 861), 12 studies from 6 countries in the Western Pacific Region (*n* > 500), 6 studies from 4 countries in the Americas (*n* > 160), 6 studies from 4 countries in the Eastern Mediterranean (*n* > 282), and 6 studies from 7 countries in the European Region (*n* > 161). The majority of studies investigated self-medication with antibiotics among community members, pharmacy customers, patients, caregivers, or more specific groups including teachers, students or community health volunteers. The remaining studies explored broader health topics, such as TB or sexually transmitted infections, in population groups including unmarried adolescents, sex workers or patients with specific illness, and self-medication with antibiotics were part of the findings. A detailed description of study characteristics is provided in Table 1.

**Table 1.** Summary study descriptions

Author, year	Location	Research focus	Qualitative methods	Setting/context	Population
**WHO African Region**					
Ackumey 2011	Ghana	Help-seeking for Buruli ulcer	Interviews	Ga-West and Ga-South municipalities in Accra	181 patients
Cremers 2013	Gabon	TB: patient perceptions and healthcare seeking	Participant observation, interviews, focus groups	Healthcare settings, patient homes and traditional healing settings	30 TB patients 36 relatives
Musheke 2013	Zambia	Self-care practices and health experiences of people living with HIV	Interviews	Low-income, high-density urban residential area of Lusaka	62 people living with HIV
Afari-Asiedu 2018 and 2020	Ghana	Community antibiotic access at household and community level	Focus group, interview	Rural Kintampo North and South Districts, Bono East Region	16 residents interviewed 16 focus groups (99 residents)
Ahiabu 2018	Ghana	How households respond to ill-health	Observations	Two communities in Eastern region in southern Ghana	12 rural households
Anstey Watkins 2019	South Africa	Access, use and understanding of antibiotics	Interviews, focus groups	Agincourt rural community	17 residents interviewed 6 focus groups (43 residents)
Kamati 2019	Namibia	Self-medication practices for children under five	Qualitative questionnaire	Tobias Hainyeko informal settlement in the Outapi Township	100 households
Owuor 2019	Kenya	Self-medication with antimicrobials after intervention	Focus groups	Nyalenda B informal settlement, Kisumu County	30 community health volunteers
Sambakunsi 2019	Malawi	Self-medication with antimicrobials	Focus groups	Lilongwe	15 residents
Torres 2019, 2020 and 2021	Mozambique	Self-medication with antibiotics and underlying influences	Interviews, focus groups	Nine private pharmacies in Maputo city	20 interviews, 1 focus group (12 pharmacy customers)
Agu 2020	Nigeria	Misconceptions about HIV/AIDS	Focus group	Ebonyi state	12 focus groups
Cambaco 2020	Mozambique	Community knowledge of antibiotics and practices	Interviews, focus group	Manhica District	16 residents interviewed 4 focus groups
Eibs 2020	Guinea-Bissau, Central African Republic, Democratic Republic of Congo and Sudan	Use of antibiotics and the main drivers within different contexts	Interviews, focus groups, case discussions, field observations	Clinic and community settings	62 residents interviewed 14 focus groups
Rodrigues 2020	Mozambique	Antibiotic consumption practices	Observation, informal conversation, focus groups and interviews	Maputo	17 residents interviewed 7 focus groups (42 residents)
Dixon 2021 (and Nayiga, 2022)	Malawi and Uganda	Patterns and reasons behind antibiotic use	Anthropological ethnographic study using observations and interviews	Chickwawa rural district in Malawi; Mbare and Budiriro suburbs of Harare; Namuwongo informal settlement of Kampala and Nagongera rural district in Uganda	1811 low-income households
Gbagbo 2021	Ghana	Self-medication practices among pregnant women	Focus group	Antenatal Care clinics in the Effutu and Agona West municipalities	6 focus groups (36 women)
Musoke 2021	Uganda	Access, use and disposal of antimicrobials	Focus groups	Kajjansi and Kasanje town councils in Wakiso district	4 focus groups (farmers)
Nabirye 2021	Uganda	Antibiotics and structural factors	Interviews, focus groups, participant observation	Urban informal settlement in Kampala, capital of Uganda	13 residents interviewed 2 focus groups (24 residents)
Davis 2022	Tanzania	Challenges of health care in relation to Antimicrobial Resistance	Interviews, focus groups	Three villages in Kilimanjaro, Arusha and Mwanza	121 agro-pastoral, pastoral and rural smallholders
Emgard 2022	Tanzania	Mothers’ experiences of antibiotic use in under-five children	Focus groups	Parents or guardians attending primary healthcare facilities in Moshi urban and rural districts	54 mothers
Machongo 2022	Malawi	Lived experiences of caregivers of children under five on antibiotic usage	Interviews	In a Zomba Central Hospital in the Children’s Ward, Zomba-Malawi	16 caregivers
Shembo 2022	Democratic Republic of Congo	Community knowledge of antibiotics and practices	Interviews	Pakadjuma urban slum, in Kinshasa	18 adult heads of households
Green 2023	Kenya, Tanzania and Uganda	Relationship between multidimensional poverty and antibiotic use	Interviews, focus groups	Kenya: Makueni, Nairobi and Nanyuki; Tanzania: Kilimanjaro, Mbeya and Mwanza; Uganda: Mbarara, Nakapiripirit and Nakasongola	82 interviews with patients, 44 focus groups with residents
Mambula 2023	Uganda and Niger	Use or demand for antibiotics	Focus groups, interviews	Four hospitals or health centres each in Mbarara, Uganda and Niamey, Niger	24 caregivers in Uganda, 30 caregivers in Niger
Valia 2023	Burkina Faso	Illness perceptions, range of healthcare providers in the community, antibiotic knowledge and reasons to seek healthcare outside healthcare facilities	Focus groups, interviews, informal conversations	Nanoro health area and Nazoanga health area	2 focus groups in Nanoro health area with 24 farmers 2 focus groups in Nazoanga health area with 24 farmers
**WHO African and South-East Asian Region**					
Do 2021	Ghana, Mozambique South Africa, Bangladesh, Thailand and Vietnam	Access and use practices across communities in six LMIC and comparison by national income status	Interviews, focus groups	Community setting	16 interviews and 4–6 focus groups in each country.
**WHO South-East Asian Region**					
Boonmongkon 2001	Thailand	Women’s explanatory models about what ailed them, forms of self-treatment, patterns of healthcare seeking, types of health services available	Ethnographic observation, interviews, focus groups	Rural, Lao-speaking (Isaan) population of Khon Kaen province, located in the impoverished Northeast	Interviews with 150 women and 20 men 3 focus groups
Chandy 2013	India	Reasons for inappropriate use of antibiotics	Focus groups	Urban and rural areas of Vellore district in the state of Tamil Nadu	2 focus groups with higher socioeconomic residents 2 focus groups with lower socioeconomic residents
Sahoo 2014	India	Community perceptions of infectious diseases, antibiotic use and antibiotic resistance	Focus groups, interviews	Community level in two districts (Khurda and Malkangiri) of Odisha	8 focus groups with 53 residents 10 interviews
Widayati 2015	Indonesia	Beliefs about using non-prescribed antibiotics	Interviews	Community level in Yogyakarta City, Indonesia	25 interviews
Kotwani 2016	India	Perceptions and knowledge about antibiotic use and resistance	Focus groups	Two private and three government schools in five municipal wards of West Delhi	10 focus groups: 1 teacher and 1 student focus group per school, 4–8 teachers per teacher focus group and 15–20 students per student focus group
Barker 2017	India	Social determinants of antibiotic use and how healthcare access, health knowledge, and income impacts patients’ antibiotic use practices	Interviews	Five villages in the northern state of Haryana, India	20 interviews with village residents
Retnaningsih 2017	Indonesia	Behaviour of female sex workers in preventing transmission of STDs and HIV	Observation, informal conversation	Localization of Gambilangu Semarang, Java province	5 female sex workers
Kaljee 2018	Nepal	Experiences of households affected by typhoid fever	Interviews, focus groups	Eleven urban and peri-urban sections in Kathmandu and Patan municipalities and two rural towns, Dhulikhel and Banepa	22 interviews with households 2 focus groups with female community health volunteers
Chowdhury 2019	Bangladesh	Practices around access and use of antibiotics and understanding of antimicrobial resistance	Interviews, focus groups	Ten villages across four different geographic areas in the economically deprived rural area, in the Chandpur District	16 interviews 6 focus groups with 43 residents
Lucas 2019	Bangladesh	How households in Bangladesh were accessing antimicrobials	Interviews	Urban area within Gazipur District and rural area within Mirzapur District	48 households (24 rural and 24 urban)
Sunpuwan 2019	Thailand	Access to and use of Yaa Chud at the community level	Interviews, focus groups	Urban and semi-urban communities of Kanchanaburi in western Thailand	16 interviews and 6 focus groups with residents
Karuniawait 2020	Indonesia	Antibiotic practice in the community	Interviews	Boyolali and Semarang	26 patients or customers attending an urban and a suburb pharmacy
Adhikari 2021	Nepal	Drivers of over-the-counter antibiotic sales	Focus groups, interview	Three provinces in western, eastern and central Nepal: tertiary hospitals and drug stores around these hospitals	8 Focus Group Discussions with 37 patients or customers 5 interviews
Kalam 2021	Bangladesh	Social drivers of antibiotic use during the COVID-19 pandemic	Interviews	Dhaka and Chattogram (both COVID epicentres)	40 interviews (20 with people diagnosed with COVID-19, 20 with people who had COVID-19 symptoms)
Kotwani 2021	India	Knowledge, practice and behaviour of consumers towards antibiotics	Interviews	Eleven districts of the National Capital Territory of Delhi	72 consumers in the community with 5–7 from each district
Nizame 2021	Bangladesh	Awareness of relevant policies and guidelines among drug shop customers	Workshops, interviews	Rural area (Kaliganj sub-district of Gazipur District) and urban area (Rupganj sub-district of Narayanganj District)	2 workshops in each rural and urban site 12 interviews with residents
Van Melle 2021	Sri Lanka	Knowledge, perceptions and attitudes regarding antibiotics and antibiotic resistance	Interviews	Tertiary care public hospital in Southern Province	18 patients with lower respiratory tract infections
Inchara 2022	India	Practices related to antibiotic use and possible reasons for these practices	Interviews	A rural tertiary health centre in Kolar	Interviews with diabetic inpatients (number not stated)
Jones 2022	Nepal	Drivers of antibiotic misuse and overuse from the perspective of the communities	Workshop, focus groups	Peri-urban site in Chandragiri Municipality and urban settlement in Bhaktapur Lockanthali	Workshops and focus group discussions with 23 residents
Sharma 2022	India	Health-seeking behaviour for childhood morbidities and concerns amongst caregivers of under-five children during the COVID-19 pandemic	Interviews	Low-income urban agglomerate in the North-East district of Delhi	17 mothers
Dhungel 2023	Nepal	If and how the pandemic posed pressure on antimicrobials	Interviews	In and around three major tertiary hospitals in Kathmandu	10 COVID-19 patients
Mitchel 2023	Nepal	Role of children in relation to antibiotic use and Antimicrobial Resistance	Secondary analysis of interview transcripts	Chandragiri peri-urban settlement in Kathmandu and Lockanthali town in Bhaktapur	10 adults
**WHO Western Pacific Region**					
Simon 1996	Philippines	Management and treatment of cough in small children	Interviews, case studies, observations	Tagbilaran City, Bohol	65 mothers and 12 grandmothers
Hoa 2007	Vietnam	Health-seeking behaviour and drug use for preschool childhood illnesses	Focus group	Hanoi city (urban) and Hatay province (rural)	6 focus groups (3 urban, 3 rural) with 49 caregivers, including fathers, mothers and grandparents
Jin 2011	China	User framings of technologies, socio-technical systems and practices	Interviews, focus groups	Four villages in Hubei and Shandong provinces (mid-socio-economic status provinces)	28 interviews with villagers, 12 focus groups including 3 with village committees and 9 with patients
Le 2011	Vietnam	Knowledge, attitudes and behaviours of parents in their use of drugs for respiratory illness or diarrhoea among children under 5 years of age	Interviews, focus groups	FilaBavi in the Bavi district (rural area)	4 focus groups with 28 mothers of children under five
Huang 2015	China	Changing social and economic environment, migration patterns, knowledge of sexually transmitted infections, risk behaviours and health beliefs	Interviews	Zhabei district of Shanghai city	16 female streetwalkers
Om 2017	Cambodia	Antibiotic-seeking behaviour in the community and drivers of antibiotic misuse	Interviews, focus groups	Multiple settings including hospitals, pharmacies and primary healthcare centres	35 interviews with patient family members
Kuijpers 2018	Cambodia	Illness interpretations, perceptions of treatment and first-line treatment practices and health-seeking behaviour associated with enteric fever	Ethnographic observation, interviews, informal conversations	Multiple settings in Phnom Penh	21 interviews with patients, 17 informal conversations
Irawati 2019	Malaysia	Community residents’ knowledge, attitudes and perceptions regarding antibiotics	Interviews	Jelutong District, Penang	22 residents
Lambert 2019 [2023]	China	Patterns of antibiotic use in rural China	Ethnographic observation, interviews	Anhui Province	59 patients and customers at retail pharmacies
Vilay 2019	Lao PDR	Knowledge, perception, and preventive and treatment behaviour regarding malaria	Focus groups, interviews	Champasak and Attapeu provinces	7 focus groups with 32 military personnel and 17 interviews with military personnel
Wang 2020	China	Determinants of non-prescription antibiotics dispensing	Interviews	Four counties from each province of Zhejiang, Hubei and Sichuan	24 residents
McKinn 2021	Vietnam	How people in Vietnam use antibiotics in community settings, and the factors that impact their practices and decision making regarding antibiotics	Interviews	One rural and one urban site in the municipality of Hanoi in the north of Vietnam, and one rural and one urban site in Ca Mau Province in the south	50 residents
**WHO Americas Region**					
Martinez 1997	Mexico	Terms used by mothers for acute respiratory infections and their management practices	Ethnographic observations, interviews	Rural communities in central Mexican highlands	24 mothers of children under 5 and 12 mothers of children who died from respiratory infections
Person 2006	Dominican Republic	Health beliefs, health-seeking behaviours and self-care practices of women with lymphoedema in filariasis-endemic areas	Interviews, focus groups, field notes and photographs	Filariasis-endemic areas of the country	56 women with lymphoedema of the leg: 28 interviews and 3 focus groups
Ruelaz Gonzalez 2012	Mexico	Medicine use by senior citizens	Interviews	Morelos	22 older adults
Cebolla Badie 2013	Argentina	Use and meaning of pharmaceuticals in indigenous communities	Interviews	Rural and peri-urban areas of Salta, Formosa and Misiones	Indigenous community resident (number not specified)
Salazar Villamarín 2016	Colombia	Maternal expectations for treatment of acute respiratory infections in children	Ethnographic, interviews	Indigenous area of south-west Columbia	50 indigenous mothers seeking medical attention for their children at a village hospital
Aponte-Gonzalez 2019	Colombia	Perceptions regarding the use of antibiotics without prescription	Focus group	Public schools (low-medium socioeconomic levels) and private companies (medium-high) in Bogotá	21 people in 4 focus groups
**WHO Eastern Mediterranean Region**					
Kandeel 2014	Egypt	Knowledge, attitudes and practices of patients regarding antibiotic use for acute respirator infections, and to identify cultural and societal determinants contributing to the use of antibiotics	Focus groups	Village of Nazlet El Fellaheen, Minya district	20 focus groups with 160 residents
Joseph 2016	Pakistan	Layperson awareness and perceptions of antimicrobial resistance	Interviews	Thirty-one settlements in central Karachi, a region with a high prevalence of diarrhoeal disease and Antimicrobial Resistance	40 residents
Atif 2019	Pakistan	Public knowledge, attitudes and practices regarding antibiotic use	Interviews	Two pharmacies in Bahawalpur, Punjab	16 interviews with survey respondents
Arabiat 2021	Jordan	Family beliefs about the causes of illness and how best to manage illness	Family group interviews	Urban and metropolitan areas	25 Arab families
Burtscher 2021	Afghanistan	Knowledge, perceptions and attitudes toward antibiotics	Interviews, group discussions	Outpatient departments of the Ahmad Shah Baba District Hospital in Kabul	21 patients/caretakers
Khan 2022	Pakistan	Knowledge, attitude and practices of antibiotic consumption and resistance	Interviews	Community pharmacies in post-conflict areas of (conflicted zones)	20 residents
**WHO European Region**					
Kaae 2017	Albania	Antibiotic knowledge, attitudes and behaviours	Interviews	No information provided	4 patients with prescription, 4 patients without prescription
Ostergaard 2018	Kyrgyzstan	Perceptions and practices among caregivers for recurrent Lower Respiratory Tract illnesses in children under 5 years	Interviews	Health clinics, one in the lowlands (Chui province) close to the capital Bishkek, and one in the highlands (Naryn province), far from the capital	13 caregivers including 11 mothers and 2 grandmothers
Jakupi 2019	Kosovo	Attitudes, experiences and knowledge of users towards antibiotics	Interviews	Capital city	4 patients with prescription, 4 patients without prescription
Kaae 2020	Armenia, Georgia, Kazakhstan, Moldova, Russia and Tajikistan	Antibiotic knowledge, attitudes and behaviours and how these may be influenced by the national healthcare systems across six countries	Interviews	Hospitals, pharmacies	40 patients with or without Antibiotics prescription
Westerling 2020	Turkey, Germany, Netherlands and Sweden	Views of Turkish citizens and Turkish migrants in Germany, the Netherlands and Sweden of policies related to rational antibiotic use	Focus groups, interviews	Clinical and community settings	3 focus group of 37 people in Turkey
Canterero-Arevalo 2022	Russia	Antibiotic practices, knowledge and attitudes	Interviews	North-west Russia	55 adults who had used Antibiotics in last 3 months

### Study quality

Thirteen studies met all seven of the quality criteria (high-quality), 49 studies met four to five of the criteria (medium-quality) and 16 studies met less than four of the criteria (low-quality) (see Table S1). In terms of privileging subjective meaning and theoretical and conceptual adequacy, only the high-quality studies investigated subjective meanings in depth and interpreted data in an inductive way. Many studies described participants’ views and practices and evaluated them from a biomedical standpoint. Most studies compared and triangulated findings between sources. Only the high-quality studies and some of the medium-quality studies gave full description of the context and researcher reflexivity.

### Translation of second-order constructs

We developed 16 translated constructs, which were combined into six overarching line-of-argument themes: experience of accessing antibiotics in the community and health services; judging risks, costs and benefits when deciding to seek antibiotics; influence of past experience of treatment and recovery; collective experience influences antibiotic use; self-medication with antibiotics is routine practice; and antibiotics as powerful medicines for symptoms of infections.

#### Experience of accessing antibiotics in the community and from health services

Participants in the majority of studies reported that over-the-counter (OTC) antibiotics were readily available from multiple local retail outlets, which included retail pharmacies, groceries, medicine shops and medicine peddlers, from where it was easy and convenient to access antibiotics.^23,25,27,46–77^ The types of retail outlet supplying antibiotics OTC varied between countries, with market stalls and street peddlers mentioned in African studies^67,78–81^ but not elsewhere, and retail pharmacies, medicine shops or shops that sold medicines alongside other goods found in all regions. The main reasons for purchasing antibiotics directly from retail outlets (instead of via a clinical consultation) was to reduce both the direct costs of treatment (cost of medical consultation and travel to clinic) and the indirect costs of lost income, which was particularly important for those with low-paid or insecure jobs.^27,47–49,53,54,56,58,59,61,67,68,70,76–78,82–90^ Retail pharmacies and other medicine sellers would allow the purchase of antibtioics in fractions of a course, or daily, or sometimes on credit, which made them more accessible to those on very low incomes.^23,47,57,63,66,91,92^

‘For fever, cough, and dysentery I go to a local drug [medicine] shop for seeking healthcare. The treatment cost is low in drug shop and it is nearby. Hospitals are far away from my area. If we get better from drug shop’s treatment, then we do not visit any doctor.’ (Narayanganj, Bangladesh)^58^

‘I myself know that I should go to the doctor when I’m ill but going to the doctor means trading time for work… It’s a dilemma. (…) If I go to the doctor this morning, I won’t have money for food this morning, meaning that my children won’t have anything to eat (…) I have to find the quickest solution to the problem (…) I choose to go to the pharmacy to buy Western medicines because it’s quicker…’ (Ca Mau, Vietnam)^49^

‘Sometimes you are sick, and you don’t have money to take you to the [clinic] and thus decide to just buy [antibiotics] thinking that you will be healed.’ (Makueni, Kenya)^93^

In contrast, participants described finding it difficult to access treatment from health facilities. Many areas had few or no government health services and people had to travel, sometimes long distances, to access them.^48,54,56,58,61,63,66,67,73,91,93–95^ When people did attend government health facilities, they faced long delays before being seen and treated, which added to the costs of lost income.^17,25,27,46,51,54,56,60,68,73,78,79,81,87,91,93,96–99^ Participants in many studies reported that clinics often lacked medicine stocks and sometimes lacked healthcare staff or testing facilities, so people were not confident that clinics could provide medical care.^17,23,46,48,54,56,58,60,63,66,67,73,92,93,96,100^ It was costly and sometimes difficult to find transport to health facilities, particularly during seasons when bad weather was more common.^48,54,61,63,66,78,79,81,91,96,101^ Insecurity was also a barrier to travelling to distant health facilities, particularly for women.^48,79^ People could not afford time away from work or from caregiver duties at home either to visit a clinic for themselves or to stay with a sick person in hospital.^57,60–62,69,85,93^

‘There is a scarcity of qualified doctors [in our locality] and there are no standard government health facilities like medical college hospitals. Also, the unavailability of medicine is a common issue in the government health facility center’ (Narayanganj, Bangladesh)^58^

‘Sometimes you go through all the queues since early morning at public health facilities and you end up being prescribed flu drugs. You wasted time and money … you have to evaluate what you feel and if it is worth going to the hospital or go direct to the pharmacy and explain!’ (Maputo, Mozambique)^102^

‘The clinic shelves are empty. You only go there to pay for a card to see a nurse who only gives you a paper to go to the pharmacy. At the pharmacy, you will find everything but the prices there will tell you to go away. To get something, you are forced to buy very cheap medicine at the market like what I do. The marketplace is now my clinic.’ (Harare, Zimbabwe)^67^

In some studies, people reported poor experiences of care in state health services, which was contrasted with more positive experiences in retail medicine outlets. In these studies participants characterized services in health facilities, particularly free ones, as unwelcoming, with patients receiving little attention or empathy, and brief interactions.^17,25,46,47,60,63,66,69,73,81,89,91,93,98,99,103^ The most frequent complaint was a lack of communication and health workers not having time to engage with patients, although some also reported staff rudeness and other behaviours that made them feel unwelcome.^46,47,60,63,66,73,81,91,93,98^ Participants in a few studies reported discrimination linked to their cultural, ethnic or religious identities.^48,63,66^

‘I would like to take treatment in government hospital, but I don’t get proper service there. They don’t do their assigned duty, neglect the patients.’ (Bangladesh)^17^

‘You can reach a health facility, and somebody talks to you rudely. If a person talks to you well, even if he does not have treatment for you, you can feel better. Instead, a health worker [says] that ‘we don’t have drugs, so you can do what you want.’ (Mbeya, Tanzania)^93^

‘Even though the treatment is free in public facilities, they do not treat well without bribe or connection. (…) We are treated with negligence. If we go to the public healthcare centres, we might end up dying without treatment.’ (Bangladesh).^63^

Although the main reason for using retail outlets was low cost and ease of access, participants in some studies also described a better interaction with staff compared with that in health facilities. Participants often did not distinguish between qualified pharmacists and other staff working in retail pharmacies or other medicine sellers. In many accounts, people were deciding what to purchase themselves, but in some cases people sought and trusted advice from people working in pharmacies and retail medicine outlets (many of whom will have had little or no formal training).^17,23,25,27,46,49,52,53,66,68,91^ The basis for this trust was the experience of advice and treatment that met patients’ expectations and the fact that these workers were part of their own community.^17,23,25,27,46,49,52,53,66,69,91^

‘I go directly tell my problem to medical store guy, such as I have a cold or a fever, and then I ask him to give me the medication for it’ (Haryana, India)^61^

‘I prefer buying medicine from a nearby drug store than going to the hospital. At the pharmacy, they ask the symptoms before they give the drugs and they are very effective.’ (Ghana)^17^

‘I take medicines from workers at pharmacy. They are experienced and we had to take just one or two tablets. We trust them that they have experience so will give right medicine.’ (Punjab, Pakistan)^91^

‘They [pharmacy salespersons] tell us how to take the medicines and we depend on this advice. If they give something wrong, they will be in trouble. They might not be MBBS doctors but they are from our area, so we trust their management.’ (Bavi, Vietnam)^60^

In a few studies, participants did express concern about the quality of the medicines received from both retail outlets and official health facilities. A few participants expressed concerns that antibiotics from market medicine sellers might be poor quality or even fake.^51,67,79^ Participants in one study believed that the antibiotics sold by retail outlets were actually more effective than those provided free by government clinics,^69^ in line with beliefs that more expensive medicine has a stronger effect.^46,60,79,86,91^

‘I was worried I might get fake antibiotics if I purchase them outside a pharmacy’ (Yogyakarta, Indonesia)^51^

The common experience was of inaccessible or unaffordable and often poorly functioning health services, which left a gap that was filled by a thriving retail sector composed of pharmacies (with or without a qualified pharmacist), and other retail outlets including market stalls and travelling medicine sellers. Most people bought their antibiotics from the latter because it was the most practical choice, but for some it was their only choice.

‘Frankly speaking, our healthcare system is totally pharmacy-based.’ (Bangladesh)^63^

‘…poor people [have] no money for rice and salt, how will they buy full course of medicine…?’ (Odisha, India).^92^

#### Judging risks, costs and benefits when deciding to seek antibiotics

When deciding whether and what treatment to seek, participants assessed the severity of and perceived threat from the condition in question.^17,23,25,27,46,47,52,53,56,58–62,72,83,86,91,95,100,101,104^ They judged whether it was severe enough to make it worth the time, difficulty and expense of visiting a healthcare facility.^17,46,56,58,60,61,89^ If the illness was not deemed sufficiently serious, it was common practice among participants initially to use home remedies or pharmaceuticals kept in the home (sometimes including antibiotics) and then, if this failed to relieve the symptoms, to visit retail pharmacies or drug shops for advice and medication, including OTC antibiotics.^56,81,86,94,100^ For illnesses that were not initially judged to be severe, visiting a healthcare facility would be the last choice.^79,86^

‘If it is a minor illness then we take it (medicine) from a chemist (pharmacist) otherwise we visit the doctor.’ (National Capital Territory, Delhi, India)^105^

Many people weighed up the perceived harms and benefits of antibiotics and used them when they felt the needs outweighed the risks; they would often reduce or stop using them as soon as symptoms disappeared to reduce harmful effects on the body.^47,49,55,63^ Very few studies reported that people viewed antibiotics as harmless.^61,106^ Only one study in Indonesia found that the commonly used antibiotic, amoxicillin, was perceived to be entirely safe for self-medication.^51^ Treatment seeking was slightly different for children. The strength and potential toxicity of antibiotics was considered riskier for children, who were seen as more vulnerable, and some parents felt it was necessary to consult a doctor before using antibiotics.^17,25,26,49,51,53,61,64,72,75,81^

People’s treatment seeking was influenced by their need to recover quickly. People expressed a need to control the illness and return to normal daily activities, often for the sake of daily survival.^25,46,47,49,50,57,75,85,88,95,100,101^ People described working through illness episodes, relying on a range of antibiotics to do so because they had to make enough money every day to pay for food for themselves and their families.^57,101^

‘…medicine has helped to promote everyday life. When you get better, you run to the market to go and work for a living. That is why we give ourselves those doses. If I take 3 tablets and I go to sell food and I do not pass out, then next week I will do the same. That means [medicines] are a part of my life.’ (Kampala, Uganda)^57^

In a small number of studies, all from African countries, the risks that came from poor living conditions were an important influence on antibiotic use.^67,103,107,108^ Contaminated water and lack of sanitation led to frequent bouts of infections and, with little hope of improving these conditions, regularly taking antibiotics was the only option.^67,103,107,108^

‘Just looking at the environment we live in, you have to know that there are a lot of germs; moreover, the environment, the water we drink, bad smells, waste everywhere…’ (Kinshasa, DRC)^103^

‘You find that you and medicine are inseparable …. You strike an agreement with medicine, you are always taking medicine. Because of the situation in your area, you cannot spend a year without medicine.’ (Nagongera, Uganda)^67,107^

#### Influence of past experience of treatment and recovery

Experience of perceived effectiveness for previous illness episodes was the main influence on people’s antibiotic understanding and practices. The most common influence was the prescription of an antibiotic by a health professional to treat a specific symptom or illness: when similar symptoms or conditions recurred, people purchased the same antibiotics again, without a prescription.^19,23,25,46,51,55–57,60,61,64,65,71,74,77,80,81,84,87,91,94,100,103,105^ The embodied experience of recovery, which was associated with taking antibiotics, reinforced belief in their effectiveness for specific symptoms.^25,49,51,60,70,78,100^ Some felt it was only necessary to see a doctor the first time that symptoms were experienced, but then you would know how to treat all subsequent illness episodes and would not need to return to see a doctor (thus avoiding the expense).^56,60,65,75^

‘When my child got sick the first time I took him to hospital. My child took drugs according to a prescription and recovered, so from then onwards, I have just gone out and bought the same drug as before.’ (Bavi, Vietnam)^60^

The experience of being prescribed or advised to use antibiotics by doctors and other health professionals contributed to an understanding of antibiotics as a rational treatment that was sanctioned by medical authorities. Participants justified their self-medication with antibiotics by citing a previous prescription for similar symptoms or illness.^23,25,51,56,57,60,64,77,81,84,91,94,105,109^

‘As for coughs, even if we go to a hospital, we know they will prescribe Bactrim or amoxicillin … So, most of the time when a person has a cough we either give amoxicillin or Bactrim’ (Lilongwe, Malawi)^81^

‘Antibiotics are a medicine that the doctor will give in all types of disease.’ (Haryana, India)^61^

Regulation and public health messaging were interpreted in light of these experiences of antibiotics as a common and sensible treatment for infection. Restrictions around access to antibiotics and messaging about the dangers of side effects or overuse of antibiotics leading to AMR were taken as confirming beliefs that antibiotics were strong and potentially dangerous medicines.^17,47,49,51,63^ However, the danger of overuse was believed to lie in toxicity or the development of bodily tolerance.^49,51,53,82,103^

#### Collective experience influences antibiotic use

People were influenced not just by their personal experience of past antibiotic treatment, but by similar experiences shared across social networks and communities. Recommendations from trusted members of people’s social networks, including family, friends, neighbours and social media, influenced beliefs about what antibiotics treat, their efficacy and appropriate help-seeking practices.^23,25,27,46,49,51–56,59,63,64,74,78,79,81,83,91,94,100,110^ People gave credence to accounts from trusted friends and family of being prescribed an antibiotic for, and recovering from, something similar;^25,91,100^ they also trusted advice from people who had some kind of medical training including veterinarians and pharmacy workers.^51,52,63,81,100^

‘In my family, my husband suggests which antibiotic is to be taken as he has a good knowledge about medicines. He used to work in a pharmacy and is involved in a pharmacy business now.’ (Chandpur, Bangladesh)^63^

Social networks also helped facilitate access to antibiotics. In contexts where regulations to restrict sales of antibiotics without prescription were enforced, antibiotics could still be purchased without a prescription from pharmacies where the customer was known or where a friend or relative worked.^47,56^ Some reported that friends working in pharmacies would allow antibiotics to be purchased on credit.^111^ Similarly, people could buy antibiotics from street medicine sellers if they were known to them or introduced by a friend.^17^

#### Self-medication with antibiotics is a routine practice

Self-medication with antibiotics was very common and was part of a wider culture of self-medication for illnesses that was seen as an ordinary practice.^17,25,50,56,75–77,83,94,99,104,112,113^ Common practices included purchasing antibiotics OTC without a prescription and storing leftover antibiotics (obtained with or without a prescription) for later use,^25,47–49,55–57,72,78,81,82,91,103,104,106,111,114,115^ or to share with family and friends,^17,46,63,79,87,96,100,104,112^ or to use as a guide for future purchases of OTC antibiotics.^46,48,87^ Most of the antibiotics purchased OTC were brand name oral medicines, some were injectable medicines, and some were part of unlabelled packets of a mixture of medicines.^50,79,84^

‘No doctor’s permission [prescription] needed. (…) People here often go to [Name]’s pharmacy and tell him to sell two ampi [ampicillin] tablets, and two tetra [tetracycline] tablets, just in case.’ (Ca Mau, Vietnam)^49^

‘We keep them so that we can use them again when we get ill.’ (South Africa)^17^

Self-medication practices varied between those with higher and lower incomes. Some more affluent people reported purchasing OTC antibiotics pre-emptively or preferred antibiotics from retail pharmacies or clinics, even where antibiotics from healthcare facilities were free.^49,79^ They had leftovers because they stopped taking medicines when feeling better,^48,55^ to avoid the harms of medicines,^47,49,55,63^ or forgot to take them.^87^ They routinely shared medicines as a matter of convenience as they were busy and lacked time to collect more.^81^ In contrast, poorer participants purchased OTC antibiotics on a day-by-day basis rather than as a full course of antibiotic treatment.^17,23,57,58,63,92,96^

‘Antibiotic medicines cost around 70 takas [$US 0.8] per day and around 150 takas [$US 1.6] for two days. I do not have any other financial support, so how can I afford this? Think about my situation. If a man cannot afford to buy milk and eggs, how can he afford to buy full doses of antibiotics?’ (Chandpur, Bangladesh)^63^

#### Antibiotics as powerful medicines for symptoms of infection

Antibiotics were mainly understood as treatments for embodied symptoms such as fever, cough and cold;^46–48,53,56,62,64,74,75,81,82,87,89,90,92,94,100^ pain caused by sores or wounds on the skin;^17,50,62,84,85,87,100,104^ internal pain or inflammation such as stomach pain;^17,46,48,50,55,62,74,78,87,90,100,104^ or serious illnesses.^53,100,116,117^ Antibiotics were not always thought of as a distinct type of medicine, but as the same, or in the same group as, other medicines that are used to treat similar symptoms or illnesses, including anti-inflammatories or painkillers, or medicines that have a similar appearance (e.g. coloured capsules).^17,25,52,56,57,78,82,89,104^ The term ‘antibiotic’ was not known or used in all contexts: in some areas of Asia, the term ‘anti-inflammatory’ was widely used to describe antibiotics and other medicines used to treat common infections;^24,82,94^ in some places (common in African countries) the specific names (e.g. amoxicillin) were known but not the general term antibiotics.^23,46,48,78,80,115^ Some people (again, mainly from African countries) distinguished types of antibiotics based on their appearance (e.g. colour of capsule) and believed they treated different symptoms or illnesses, but others viewed them as interchangeable.^17,46,78^

‘It’s our own thinking; you know that when you are experiencing pains in your stomach, it is sore that has developed in your stomach so you can take the tetracycline or the white antibiotic. This is because it’s the same way that, when the sore develops on your body, you will use same tetracycline or white antibiotic.’ (Bono East Region of Ghana)^78^

Antibiotics were universally thought of as ‘big medicines’ that are effective, powerful and provide quick relief from symptoms.^17,25,46–49,52,55,56,61,63,75,79,84,86,90–92,100,106^ This was based on bodily experiences of symptom relief and recovery following the use of antibiotics.^49,52,55,61,79,91,100,118^ Many believed that antibiotics worked by cleaning toxins or dirt from the body and supporting the immune system, thereby strengthening the body’s ability to fight illness.^55,61,79,89,100,110^ Therefore, antibiotics were perceived as a sensible treatment for any severe illnesses or symptoms regardless of cause; this included non-infectious conditions such as hypertension and lethargy as well as potentially serious viral infections like COVID and HIV.^63,100,117^

‘COVID-19 is not a bacterial disease; rather it is viral. Antibiotics do not work for viral disease but help to improve immunity. For this reason, I used antibiotics. You know, coronavirus impacts on whole body. Body cannot fight against the virus but if your immune system is strong then your body can fight against further damage caused by COVID-19.’ (Dhaka and Chattogram, Bangladesh)^100^

Antibiotics were identified as ‘Western medicines’, which were believed to have a powerful effect on the body. This powerful effect would speed recovery but could also cause harm.^17,25,49,52,56,60,63,86,94^ The more antibiotics were taken (higher doses or longer course), the greater the harm.^49,53,55,60,86,92^ Imported and more expensive drugs were reported to have a stronger effect and do less harm.^46,60,79,86,91^ The harm was thought to be caused by the toxicity of antibiotics ‘artificially made with chemicals’.^49,51,100^ In contrast, traditional herbal medicines (often used first or alongside antibiotics) were understood to have a gentler effect and to be much safer.^60,94^

‘Flowers and leaves are not harmful, but Western drugs [medicines] are harmful’ (Bavi District, Vietnam)^60^

‘But at the same time it’s also worse because after taking this medicine, some problems may happen. The body will become weak because it has a lot of power. … The body will become weak, the head dizzy, and someone can face vomiting problems. So many problems may happen.’ (Gazipur and Mirzapur Districts, Bangladesh)^52^

The term ‘antibiotic resistance’ was rarely known; there was some awareness of an issue but it was usually taken to mean that the body developed resistance or tolerance to antibiotics as a result of excessive use, often necessitating the use of different, stronger medicines.^25,49,51,53,56,74,80,82,89,91,92,104,119^ A few people made a link to ideas of environmental degradation: they believed that some antibiotics were no longer effective because bodily immunity had been weakened due to chemicals in food production, climate change or pollution.^91,92^

‘The doctor said that if we gave the antibiotics four or five times per year, our son would produce defences against the antibiotics, and he would need a stronger one. If the boy gets used to the antibiotic, he would need a higher level.’ (Bogotà, Colombia)^25^

‘Gradually, immunity power of our body is decreasing; for that reason we are requiring high doses of medicines, i.e. instead of one dose there is requirement of two doses. We are applying more chemicals in our foods. Our food habits and climate, both are changing. Due to these reasons body immunity is decreasing and medicines are not working.’ (Khurda, India)^92^

## Discussion

To our knowledge, our study is the first systematic review to synthesize the qualitative evidence on the use of antibiotics without prescription in LMIC contexts. Difficulties in accessing official health services (including time costs) and poor experiences of care from these services were also key factors in people’s decision to buy antibiotics without prescriptions. For many people, it was easier to access treatment from a retail pharmacy than from a qualified medical practitioner. However, the potential for harm and the actual cost of OTC antibiotics meant that people would weigh up the risks, costs and benefits before deciding whether to use antibiotics, with perceived illness severity being a key trigger for antibiotic use. The common experience (individually and collectively) of health professional antibiotic prescribing promoted the idea of antibiotics as sanctioned by medical authorities to treat certain illnesses. Antibiotics were understood as powerful medicines to treat embodied symptoms such as fever, cough, pain and sometimes serious illnesses in general. This was based on collective experience of antibiotics being prescribed for illnesses with these types of symptoms, people’s bodily experiences of symptom recovery and the perception of antibiotics as a ‘Western medicine’, which carried connotations of being both a strong therapeutic agent and a potentially harmful one. Buying and using antibiotics without prescription was seen by most people as a normal health care practice.

**Figure 2.**
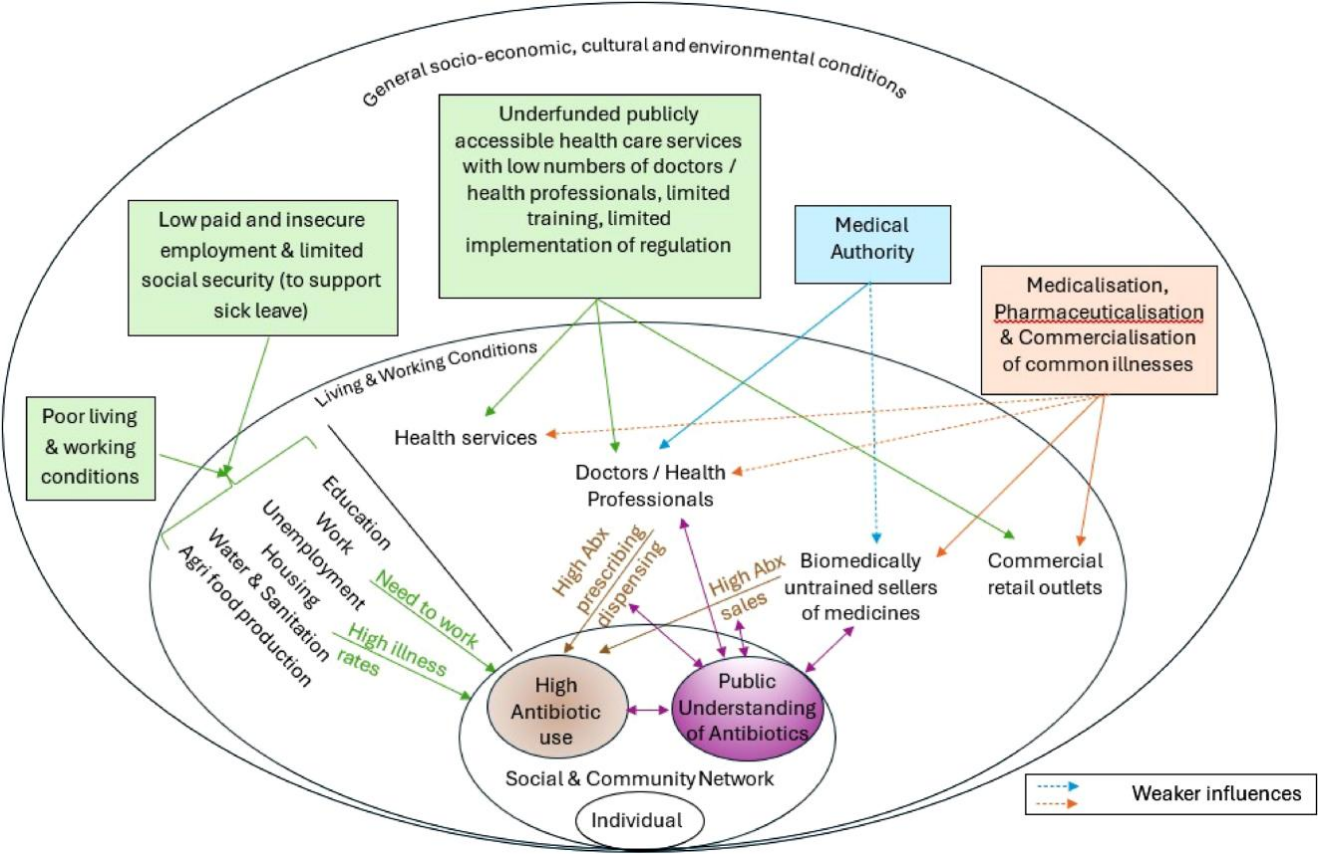
Proposed model of influences on antibiotic self-medication in LMIC. Abx, antibiotics.

Our synthesis of research findings identified influences that are variously operating at the level of wider societal context (e.g. poor socioeconomic conditions affecting both formal health services and individuals), between communities and the healthcare available to them (the bidirectional influences of doctors and medicine sellers) and within social networks (collective experience and shared beliefs). Dahlgren and Whitehead’s^120^ socio-ecological model of health determinants provides a suitable framework to depict the integration of these influences operating at individual, community and wider socioeconomic levels. Following on from the thematic synthesis presented in the results and considering how these influences relate both to one another and to influences already identified in the wider literature (e.g. commercial drivers of OTC antibiotic sales), we propose a model of how these influences operate together to promote overuse of antibiotics (Figure 2).’

The wider socioeconomic conditions in LMIC influence antibiotic use through several routes. The poor living conditions that affect the majority of LMIC populations contribute to high illness rates, including high rates of infections.^121^ Illness makes it difficult to work, which is particularly problematic for those in low-paid or insecure work, and although there are schemes to support income security during illness in most LMIC, coverage is low and often excludes informal and low-income workers.^122^ This means that many feel pressure to keep working or return to work quickly, and they use antibiotics in the belief that they support quick recovery.^23,25,50,57,67,75,88,101^ In this way, antibiotics function as a ‘quick fix’ for constant illness caused by poor living environments,^57^ in turn caused by wider structural and political issues in LMIC contexts.^20,34^

Limited state funding for healthcare services and for regulation implementation contributes to the high rates of antibiotic sales without prescription. In most LMIC, locally available therapeutic approaches include state-funded and private biomedical services, formal non-biomedical therapeutic practitioners (e.g. ayurvedic or traditional Chinese medicine), traditional healers, and commercial sellers (retail pharmacies, medicine shops and sellers) of treatments including both biomedical and traditional.^123–125^ This is sometimes termed a medically pluralistic system, although this conceals considerable complexity and the important distinction between practices that are sanctioned by the state and those that are not.^123,126^ State-funded biomedical healthcare services vary considerably in the quality of care both between and within LMIC.^127,128^ In many settings healthcare is not so much provided by the state as purchased by individuals from a range of providers.^127^ This includes purchasing not just antibiotics but many other medicines OTC without prescription.^129^ Indeed, retail pharmacies can be part of an official strategy for fulfilling the aspiration of universal healthcare in LMIC.^130,131^ Although most countries have legal regulations that restrict the sale of antibiotics without prescription, these are often poorly enforced due to limited funding.^132^ However, underenforcement may also be part of a semi-official strategy to support healthcare access for populations with limited access to government health services.^12^ Purchasing antibiotics from pharmacies is a rational strategy to cope with the chronic issue of difficult access to health services.^21,67^

The authority of medical practitioners over treatment of diseases means that their antibiotic prescribing practices are socially influential, promoting high antibiotic use even when most of this use happens without the direct involvement of a qualified medical practitioner. Antibiotic prescribing rates are high for common infections in LMIC,^4^ and this review found that experience of this is the main justification given for self-medication with antibiotics for similar illnesses. Overprescription by biomedically qualified doctors contributes to unnecessary antibiotic use globally.^133^ The key issue is inherent clinical uncertainty, which means that even well-trained clinicians overprescribe antibiotics because it is very difficult to determine which infections are bacterial from symptoms alone or even from currently available tests.^134^ Overprescribing of antibiotics by biomedical doctors is also influenced by a range of issues including norms of practice, perceived patient pressure and external pressures on practice (e.g. time constraints).^133^ In LMIC, there are a wide range of practitioners (with varied training) and drug sellers supplying antibiotics;^127^ in the evidence included in this review, people rarely distinguished between (and were probably unaware of) those with different degrees of training, for example trained pharmacists versus sales assistants working in retail pharmacies. All those working in formal and informal health services may be perceived by the public to hold a degree of medical authority and thus their practice reinforces the idea of antibiotics as a medically sanctioned treatment. This creates a reinforcing feedback loop between the widespread practice of frequent antibiotic prescribing and sales and the public understanding of antibiotics. The idea of antibiotics as powerful medicines for treating common infections is a social construction that both influences and is influenced by antibiotic prescription and retailing practices by formal and informal healthcare services. This happens not at the individual level but within social networks locally, nationally and likely internationally.

The management of common infections has been subject to medicalization,^135^ pharmaceuticalization^23^ and commodification,^136^ processes that promote the consumption of antibiotics.^23^ In the fee-for-service healthcare landscapes of most LMIC, marketization and profit motives influence antibiotic provision.^6,34^ Financial incentives have an influence across the health sector through a range of mechanisms including direct payments for treatment, reimbursement from insurance schemes, and financial incentives from pharmaceutical companies.^34,137,138^ In retail pharmacies, the need to ensure the business stays in profit is prioritized over healthcare responsibilities, and regulations may be disregarded in order to retain customers.^6^ Current global strategies for improving universal access to healthcare in LMIC advocate incorporating retail pharmacies and medicine shops into national healthcare delivery systems.^11,131^ The tension between trying to implement restrictions on clinically unnecessary use of antibiotics, while also trying to improve access to adequate healthcare by incorporating profit-oriented businesses, is rooted in the limitations of underfunded health systems in LMIC.^13^

### Strengths and limitations

As with all reviews, the synthesis is limited by the quantity and quality of the available evidence base. The search strategy included databases covering medical, LMIC and some anthropological literature. There may be some relevant anthropological and sociological literature published in monographs or book chapters that are not always well represented in databases and may have been missed by the search methods. The large number of studies identified, and common issues described, gives confidence that the phenomena reported here are real across LMIC, while acknowledging that there may be more to discover.

Most of the included studies came from African or Asian countries, with some studies from Eastern Europe and a few from South America and the Middle East. The commonality of main issues identified across the studies, and the resonances with similar research findings in HICs, gives confidence in the transferability of findings more widely. However, by its nature, an evidence synthesis highlights commonalities and hence may underplay or obscure certain features that are distinctive within specific regions or countries due to differing sociocultural characteristics and/or health systems. In addition, caution should be used when applying these findings to contexts that are underrepresented in the primary studies, notably Central and South American LMIC.

Most included studies were high to moderate quality. The main quality issues were underdeveloped themes,^139^ and studies that had been conducted through a biomedical lens that failed to identify lay conceptions of illness and treatment.^43^ Since high- and medium-quality papers reported ample primary data, it was possible to translate meaning across papers drawing on both authors’ theme descriptions and primary data together. Quality appraisal was used to inform the order in which studies were incorporated into the synthesis, such that the most robust studies were the most influential. The major themes reported in this synthesis were all supported by themes identified in the more robust studies and ample primary data across all studies.

## Conclusions

Previous studies have criticized AMS strategies for their focus on individual behaviour change while ignoring wider systemic influences.^20,34^ This review and resulting model illustrates why focusing on the individual level means fighting an uphill battle against the wider social and systemic pressures that drive antibiotic use. Public health messaging is a weak influence as long as high antibiotic prescribing and selling rates continue to confirm public understanding of antibiotics as an effective treatment sanctioned by medical authority. The practice of health professionals is thus an important point of intervention, even in contexts where antibiotics are easily accessible from community sources. The design of AMS campaigns should acknowledge and make use of the continuous knowledge exchange that occurs between health providers and lay publics, rather than treating these sectors as autonomous.

Community medicine retail outlets are another crucial point of intervention to reduce unnecessary use of antibiotics in LMIC. Interventions to improve antibiotics dispensing (ensuring access while reducing overuse) from private medicine sellers in LMIC have had mixed success.^140–142^ The most difficult challenge to overcome is the financial incentive since people in these roles own or work for businesses that profit from selling medication. However, an intervention with medicine sellers and pharmacy staff in East Africa that combined training with a business model to ensure overall profitability, reported success in reducing antibiotic use.^143^ Where strategies for universal healthcare include retail pharmacies, these need to have integrated AMS strategies, so that the survival of these (often small) businesses is not dependent on selling antibiotics that are often not needed. This review found that people consider the potential harms and costs of antibiotics before deciding to self-medicate and if they could be convinced that there is no benefit from taking antibiotics for certain conditions (such as common mild respiratory tract infections), this might help to reduce use.

AMS in LMIC needs to be integrated into wider plans for delivery of universal healthcare to give people better quality care, which includes more appropriate antibiotic use by reducing unnecessary use but providing access to these essential drugs when needed. This means tailoring AMS interventions to each setting because of the context-specific variability in configurations of health systems and associated treatment seeking practices. The model outlined here uses the pre-existing socio-ecological model^120^ to propose how the main findings from this review relate to each other and to already known common drivers (e.g. commercial influences on supply). Further research using a systems thinking approach is needed to interrogate the model proposed here and to develop interventions that address the relevant drivers of antibiotic use in specific settings.

## Supplementary Material

dlae165_Supplementary_Data
