# Supplementary material for: Influences on use of antibiotics without prescription by the public in low- and middle-income countries: a systematic review and synthesis of qualitative evidence
Source: JAC Antimicrob Resist. 2024 Oct 25;6(5):dlae165. doi: 10.1093/jacamr/dlae165 (PMC11503652; doi:10.1093/jacamr/dlae165)
Supplement: dlae165_Supplementary_Data [file dlae165_supplementary_data.zip › Supplementary file Protocol.docx]

**S1: Protocol for a systematic review of qualitative evidence ‘Influences on use of antibiotics without prescription by the public in low- and middle-income countries’**

**Research aim**

To investigate the perceptions and practices of people living in LMIC that influence their use of antibiotics without prescription to self-treat illness.

**Methods**

We will follow methods set out in the Cochrane handbook(1) for searching, screening and data extraction. We will follow the meta-ethnographic method for synthesizing qualitative evidence.(2, 3) We will follow the preferred Reporting Items for Systematic Reviews and Meta-Analyses (PRISMA) guidelines.

SPICE for qualitative review

**Setting**: LMICs

**Perspective**: from the perspective of non-medically trained people, including the general public, patients, and care givers.

**Phenomenon of Interest**: anything that influences consumption antibiotics without prescription including beliefs about antibiotics and antibiotic resistance, cultural norms and practices, financial influences at individual and societal level, health delivery systems, regulations and enforcement.

**Comparison:** None

**Evaluation**: Qualitative or mixed methods study types that use methods including ethnographic, semi-structured or in-depth interviews, or focus group discussions.

Types of study to be included

Qualitative studies

Exclusion criteria for QES

1. Studies not reporting data from LMIC settings
2. Studies reporting views of those who have received medical training including biomedical doctors and medical students, nurses, pharmacists or other biomedical health professionals
3. Studies reporting views of those employed in clinical settings including hospitals, private clinics offering biomedical treatment, village clinics and retail pharmacies or drug (medicine) shops
4. Studies not reporting data on phenomena of interest
5. Studies about non-human antibiotic use
6. Studies not reporting qualitative data

Search strategy

We will search health science database and databases that specialize in LMIC health literature:

- MEDLINE
- EMBASE
- CINHAL
- WHOLIS (World Health Organization Library Database)
- GIM (Global Index Medicus)
- LILACS (Latin American and Caribbean Health Sciences Literature)
- Anthropology Plus

Search strategies will be tailored for each database and will consist of:

[Terms for antibiotic: antibiotic* OR antimicrobial* OR antibacterial* OR antimicrobial resistance OR antimicrobial stewardship] AND [Terms for consumption: self-medication OR self-treatment OR left over OR over the counter OR non-prescription OR consum* OR purchas* OR prescri*] AND [Terms for qualitative study types: qualitative OR mixed method* OR interview* OR perspective*] (see detailed search strategies for Medline, Embase and CINHAL in appendix 1)

Separate searches will be run and then the search results combined and de-duplicated before screening all results for papers relevant to review.

Study selection

Screening will be conducted in two phases. Two researchers will independently screen at least 10% of the titles and abstracts for eligibility for qualitative review. Once there is consensus on the type of studies to include in the full text screening, the rest of the titles and abstracts will be screened by one researcher. Full text versions of all papers identified through the initial screening will be obtained. Two researchers will independently screen at least 25% of these papers in 3 stages, with any disagreements resolved through discussion until consensus is reached on inclusion decisions. One researcher will screen the remaining papers for inclusion.

Data extraction

A data extraction form will be designed for the review. Contextual details and findings relevant to the phenomena of interest will be extracted, including secondary constructs (e.g. themes) with corresponding primary constructs (quotes from study participants).

Quality appraisal

The quality of papers will be assessed using the tool developed by Popay.(4) This tool has seven quality criteria and one criteria assessing relevant to policy. The seven quality criteria will be used to assess the included studies.

Synthesis

The findings will be synthesized using a meta-ethnographic approach.(2, 3) The first and second order constructs from the included studies will be extracted. First order constructs are the meanings contained with the quotes from participants in the included studies and second order constructs are the interpretations of the study authors of the data they collected, usually presented as themes or sub-themes. First and second order constructs from the same study will be kept together and translated across studies. The process of translation involves grouping concepts with similar meaning together, starting with one paper and comparing constructs from each paper in turn, in a process that is similar to the constant comparative method used in grounded theory.(2) Constructs from different studies represent interpretations of the same phenomena or different aspects of the same phenomena that can be combined through this process to produce a new interpretation of the evidence as a whole, i.e. third order constructs. These third order constructs are the interpretive ‘themes’ of the meth-ethnographic synthesis. There are different approaches to the order in which study constructs are integrated into the translation, in this synthesis we will identify those studies which are highest quality and incorporate constructs from those studies first, so that constructs from the most robust studies shape the early development of the third order constructs.

Comment on the use of the socio-ecological model in the discussion

The Dahlgreen and Whitehead socio-ecological model(5) was used in the discussion to show how the main findings related to each other and to what is already known.  It was not used in the production of the findings and the development of a model was not planned in advance.  Rather, after the synthesis was complete, at the point of discussing the meaning of the findings in relation to what was already known, the socio-ecological model appeared to be a good framework for showing how the findings related to each other and other literature in a whole system.  This was because within the inductive findings, there were influences that were working on different levels: the inter-personal, the community and the wider socio-economic and cultural level.  Therefore, we used the socio-ecological model in the discussion to help illustrate how the different influences operate together.

**References**

1. Noyes J, Booth A, Cargo M, Flemming K, Harden A, Harris J, et al. Chapter 21: Qualitative evidence. In: Higgins JPT TJ, Chandler J, Cumpston M, Li T, Page MJ, Welch VA, editor. Cochrane Handbook for Systematic Reviews of Interventions version 63 <www.training.cochrane.org/handbook>: Cochrane Collaboration; 2022.

2. France EF, Uny I, Ring N, Turley RL, Maxwell M, Duncan EA, et al. A methodological systematic review of meta-ethnography conduct to articulate the complex analytical phases. BMC medical research methodology. 2019;19(1):1-18.

3. Noblit GW, Hare RD, Hare RD. Meta-ethnography: Synthesizing qualitative studies: sage; 1988.

4. Popay J, Rogers A, Williams G. Rationale and standards for the systematic review of qualitative literature in health services research. Qual Health Res. 1998;8(3):341-51.

5. Dahlgren G, Whitehead M. The Dahlgren-Whitehead model of health determinants: 30 years on and still chasing rainbows. Public health. 2021;199:20-4.

**Appendix 1: Main Search Strategies**

**Ovid (Medline and Embase)**

| 1 | "antibiotic*".ab,kf,ti. |
| --- | --- |
| 2 | "antimicrobial*".ab,kf,ti. |
| 3 | "antibacterial*".ab,kf,ti. |
| 4 | "antimicrobial resistance".ab,kf,ti. |
| 5 | "antimicrobial stewardship".ab,kf,ti. |
| 6 | antibiotics.ab,kf,ti. |
| 7 | "antibiotic resistance".ab,kf,ti. |
| 8 | AMR.ab,kf,ti. |
| 9 | AMS.ab,kf,ti. |
| 10 | "self-medication".ab,kf,ti. |
| 11 | "self medication".ab,kf,ti. |
| 12 | "self-medicate".ab,kf,ti. |
| 13 | "self medicate".ab,kf,ti. |
| 14 | "self-treatment".ab,kf,ti. |
| 15 | "self treatment".ab,kf,ti. |
| 16 | "self-treat".ab,kf,ti. |
| 17 | "self treat".ab,kf,ti. |
| 18 | "left over".ab,kf,ti. |
| 19 | "over the counter".ab,kf,ti. |
| 20 | "OTC".ab,kf,ti. |
| 21 | "consump*".ab,kf,ti. |
| 22 | "non-prescription".ab,kf,ti. |
| 23 | "without prescription".ab,kf,ti. |
| 24 | "without a prescription".ab,kf,ti. |
| 25 | "purchas*".ab,kf,ti. |
| 26 | access.ab,kf,ti. |
| 27 | "belief*".ab,kf,ti. |
| 28 | "view*".ab,kf,ti. |
| 29 | "perception*".ab,kf,ti. |
| 30 | (knowledge adj attitude* adj practice*).ab,kf,ti. |
| 31 | (knowledge adj attitude* adj behaviour*).ab,kf,ti. |
| 32 | (knowledge adj attitude* adj behavior*).ab,kf,ti. |
| 33 | qualitative.ab,kf,ti. |
| 34 | "qualitative research".ab,kf,ti. |
| 35 | "interview*".ab,kf,ti. |
| 36 | "perspective*".ab,kf,ti. |
| 37 | "in-depth".ab,kf,ti. |
| 38 | "semi-structured".ab,kf,ti. |
| 39 | ethnographic.ab,kf,ti. |
| 40 | "focus group".ab,kf,ti. |
| 41 | "key informant".ab,kf,ti. |
| 42 | "topic guide".ab,kf,ti. |
| 43 | "thematic analysis".ab,kf,ti. |
| 44 | "phenomonolog*".ab,kf,ti. |
| 45 | "grounded theory".ab,kf,ti. |
| 46 | narrative.ab,kf,ti. |
| 47 | NVivo.ab,kf,ti. |
| 48 | MAXQDA.ab,kf,ti. |
| 49 | "mixed method*".ab,kf,ti. |
| 50 | "ATLAS ti".ab,kf,ti. |
| 51 | 1 or 2 or 3 or 4 or 5 or 6 or 7 or 8 or 9 |
| 52 | 10 or 11 or 12 or 13 or 14 or 15 or 16 or 17 or 18 or 19 or 20 or 21 or 22 or 23 or 24 or 25 or 26 or 27 or 28 or 29 or 30 or 31 or 32 |
| 53 | 33 or 34 or 35 or 36 or 37 or 38 or 39 or 40 or 41 or 42 or 43 or 44 or 45 or 46 or 47 or 48 or 49 or 50 |
| 54 | 51 and 52 and 53 |

**CINAHL**

| S1 | TI "antibiotic*" OR AB "antibiotic*" OR MW "antibiotic*" |
| --- | --- |
| S2 | TI "antimicrobial*" OR AB "antimicrobial*" OR MW "antimicrobial*" |
| S3 | TI "antibacterial*" OR AB "antibacterial*" OR MW "antibacterial*" |
| S4 | TI "antimicrobial resistance" OR AB "antimicrobial resistance" OR MW "antimicrobial resistance" |
| S5 | TI antibiotics OR AB antibiotics OR MW antibiotics |
| S6 | TI "antibiotic resistance" OR AB "antibiotic resistance" OR MW "antibiotic resistance" |
| S7 | TI AMR OR AB AMR OR MW AMR |
| S8 | TI "antimicrobial stewardship" OR AB "antimicrobial stewardship" OR MW "antimicrobial stewardship" |
| S9 | TI AMS OR AB AMS OR MW AMS |
| S10 | S1 OR S2 OR S3 OR S4 OR S5 OR S6 OR S7 OR S8 OR S9 |
| S11 | TI "self-medication" OR AB "self-medication" OR MW "self-medication" |
| S12 | TI "self medication" OR AB "self medication" OR MW "self medication" |
| S13 | TI "self-medicate" OR AB "self-medicate" OR MW "self-medicate" |
| S14 | TI "self medicate" OR AB "self medicate" OR MW "self medicate" |
| S15 | TI "self-treatment" OR AB "self-treatment" OR MW "self-treatment" |
| S16 | TI "self treatment" OR AB "self treatment" OR MW "self treatment" |
| S17 | TI "self-treat" OR AB "self-treat" OR MW "self-treat" |
| S18 | TI "self treat" OR AB "self treat" OR MW "self treat" |
| S19 | TI "left over" OR AB "left over" OR MW "left over" |
| S20 | TI "over the counter" OR AB "over the counter" OR MW "over the counter" |
| S21 | TI "OTC" OR AB "OTC" OR MW "OTC" |
| S22 | TI "consump*" OR AB "consump*" OR MW "consump*" |
| S23 | TI "non-prescription" OR AB "non-prescription" OR MW "non-prescription" |
| S24 | TI "without prescription" OR AB "without prescription" OR MW "without prescription" |
| S25 | TI "without a prescription" OR AB "without a prescription" OR MW "without a prescription" |
| S26 | TI "purchas*" OR AB "purchas*" OR MW "purchas*" |
| S27 | TI access OR AB access OR MW access |
| S28 | TI "belief*" OR AB "belief*" OR MW "belief*" |
| S29 | TI "view*" OR AB "view*" OR MW "view*" |
| S30 | TI "perception*" OR AB "perception*" OR MW "perception*" |
| S31 | TI "knowledge attitude* practice*" OR AB "knowledge attitude* practice*" OR MW "knowledge attitude* practice*" |
| S32 | TI "knowledge attitude* behaviour*" OR AB "knowledge attitude* behaviour*" OR MW "knowledge attitude* behaviour*" |
| S33 | TI "knowledge attitude* behavior*" OR AB "knowledge attitude* behavior*" OR MW "knowledge attitude* behavior*" |
| S34 | S11 OR S12 OR S13 OR S14 OR S15 OR S16 OR S17 OR S18 OR S19 OR S20 OR S21 OR S22 OR S23 OR S24 OR S25 OR S26 OR S27 OR S28 OR S29 OR S30 OR S31 OR S32 OR S33 |
| S35 | TI qualitative OR AB qualitative OR MW qualitative |
| S36 | TI "qualitative research" OR AB "qualitative research" OR MW "qualitative research" |
| S37 | TI "interview*" OR AB "interview*" OR MW "interview*" |
| S38 | TI "perspective*" OR AB "perspective*" OR MW "perspective*" |
| S39 | TI "in-depth" OR AB "in-depth" OR MW "in-depth" |
| S40 | TI "semi-structured" OR AB "semi-structured" OR MW "semi-structured" |
| S41 | TI ethnographic OR AB ethnographic OR MW ethnographic |
| S42 | TI "focus group" OR AB "focus group" OR MW "focus group" |
| S43 | TI "key informant" OR AB "key informant" OR MW "key informant" |
| S44 | TI "topic guide" OR AB "topic guide" OR MW "topic guide" |
| S45 | TI "thematic analysis" OR AB "thematic analysis" OR MW "thematic analysis" |
| S46 | TI "phenomonolog*" OR AB "phenomonolog*" OR MW "phenomonolog*" |
| S47 | TI "grounded theory" OR AB "grounded theory" OR MW "grounded theory" |
| S48 | TI narrative OR AB narrative OR MW narrative |
| S49 | TI NVivo OR AB NVivo OR MW NVivo |
| S50 | TI MAXQDA OR AB MAXQDA OR MW MAXQDA |
| S51 | TI "mixed method*" OR AB "mixed method*" OR MW "mixed method*" |
| S52 | TI "ATLAS ti" OR AB "ATLAS ti" OR MW "ATLAS ti" |
| S53 | S35 OR S36 OR S37 OR S38 OR S39 OR S40 OR S41 OR S42 OR S43 OR S44 OR S45 OR S46 OR S47 OR S48 OR S49 OR S50 OR S51 OR S52 |
| S54 | S10 AND S34 AND S53 |
