# Supplementary material for: Influences on use of antibiotics without prescription by the public in low- and middle-income countries: a systematic review and synthesis of qualitative evidence
Source: JAC Antimicrob Resist. 2024 Oct 25;6(5):dlae165. doi: 10.1093/jacamr/dlae165 (PMC11503652; doi:10.1093/jacamr/dlae165)
Supplement: dlae165_Supplementary_Data [file dlae165_supplementary_data.zip › Supplementary material Record of quality appraisal.docx]

S2: Record of quality appraisal assessment (green indicates that study was judge high quality with respect to the criteria identified in column heading).

| **Study ID** | **Overall study quality judgement**   (High / Medium / Low) | **The Primary Marker: privileging subjective meaning** | **Evidence of responsiveness to social context and flexibility of design** | **Evidence of purposeful sampling** | **Evidence of adequate description** | **Evidence of data quality** | **Evidence of theoretical and conceptual adequacy** | **Potential for assessing typicality** |
| --- | --- | --- | --- | --- | --- | --- | --- | --- |
| Afari-Asiedu 2018 | High |  |  |  |  |  |  |  |
| Arabiat 2021 | High |  |  |  |  |  |  |  |
| Cebolla Badie 2013 | High |  |  |  |  |  |  |  |
| Dixon 2021 | High |  |  |  |  |  |  |  |
| Eibs 2020 | High |  |  |  |  |  |  |  |
| Jin 2011 | High |  |  |  |  |  |  |  |
| Kalam 2021 | High |  |  |  |  |  |  |  |
| Lambert 2019 | High |  |  |  |  |  |  |  |
| McKinn 2021 | High |  |  |  |  |  |  |  |
| Rodrigues 2020 | High |  |  |  |  |  |  |  |
| Simon 1996 | High |  |  |  |  |  |  |  |
| Sunpuwan 2019 | High |  |  |  |  |  |  |  |
| Torres 2019 | High |  |  |  |  |  |  |  |
| Ackumey 2011 | Medium |  |  |  |  |  |  |  |
| Adhikari 2021 | Medium |  |  |  |  |  |  |  |
| Ahiabu 2018 | Medium |  |  |  |  |  |  |  |
| Anstey Watkins 2019 | Medium |  |  |  |  |  |  |  |
| Aponte-Gonzalez 2019 | Medium |  |  |  |  |  |  |  |
| Atif 2019 | Medium |  |  |  |  |  |  |  |
| Barker 2017 | Medium |  |  |  |  |  |  |  |
| Boonmongkon 2001 | Medium |  |  |  |  |  |  |  |
| Burtscher 2021 | Medium |  |  |  |  |  |  |  |
| Cambaco 2020 | Medium |  |  |  |  |  |  |  |
| Canterero-Arevalo 2022 | Medium |  |  |  |  |  |  |  |
| Chowdhury 2019 | Medium |  |  |  |  |  |  |  |
| Davis 2022 | Medium |  |  |  |  |  |  |  |
| Dhungel 2023 | Medium |  |  |  |  |  |  |  |
| Do 2021 | Medium |  |  |  |  |  |  |  |
| Emgard 2022 | Medium |  |  |  |  |  |  |  |
| Green 2023 | Medium |  |  |  |  |  |  |  |
| Hoa 2007 | Medium |  |  |  |  |  |  |  |
| Huang 2015 | Medium |  |  |  |  |  |  |  |
| Jakupi 2019 | Medium |  |  |  |  |  |  |  |
| Jones 2022 | Medium |  |  |  |  |  |  |  |
| Joseph 2016 | Medium |  |  |  |  |  |  |  |
| Kaae 2020 | Medium |  |  |  |  |  |  |  |
| Kaljee 2018 | Medium |  |  |  |  |  |  |  |
| Karuniawait 2020 | Medium |  |  |  |  |  |  |  |
| Kotwani 2016 | Medium |  |  |  |  |  |  |  |
| Kotwani 2021 | Medium |  |  |  |  |  |  |  |
| Kuijpers 2018 | Medium |  |  |  |  |  |  |  |
| Le 2011 | Medium |  |  |  |  |  |  |  |
| Lucas 2019 | Medium |  |  |  |  |  |  |  |
| Machongo 2022 | Medium |  |  |  |  |  |  |  |
| Mambula 2023 | Medium |  |  |  |  |  |  |  |
| Martinez 1997 | Medium |  |  |  |  |  |  |  |
| Mitchell 2023 | Medium |  |  |  |  |  |  |  |
| Musheke 2013 | Medium |  |  |  |  |  |  |  |
| Musoke 2021 | Medium |  |  |  |  |  |  |  |
| Nabirye 2021 | Medium |  |  |  |  |  |  |  |
| Nizame 2021 | Medium |  |  |  |  |  |  |  |
| Om 2017 | Medium |  |  |  |  |  |  |  |
| Person 2006 | Medium |  |  |  |  |  |  |  |
| Ruelaz Gonzalez 2012 | Medium |  |  |  |  |  |  |  |
| Sahoo 2014 | Medium |  |  |  |  |  |  |  |
| Salazar Villamarín 2016 | Medium |  |  |  |  |  |  |  |
| Sambakunsi 2019 | Medium |  |  |  |  |  |  |  |
| Sharma 2022 | Medium |  |  |  |  |  |  |  |
| Shembo 2022 | Medium |  |  |  |  |  |  |  |
| Valia 2023 | Medium |  |  |  |  |  |  |  |
| Vilay 2019 | Medium |  |  |  |  |  |  |  |
| Widayati 2015 | Medium |  |  |  |  |  |  |  |
| Agu 2020 | Low |  |  |  |  |  |  |  |
| Chandy 2013 | Low |  |  |  |  |  |  |  |
| Cremers 2013 | Low |  |  |  |  |  |  |  |
| Gbagbo 2021 | Low |  |  |  |  |  |  |  |
| Inchara 2022 | Low |  |  |  |  |  |  |  |
| Kaae 2017 | Low |  |  |  |  |  |  |  |
| Kamati 2019 | Low |  |  |  |  |  |  |  |
| Irawati 2019 | Low |  |  |  |  |  |  |  |
| Kandeel 2014 | Low |  |  |  |  |  |  |  |
| Khan 2022 | Low |  |  |  |  |  |  |  |
| Ostergaard 2018 | Low |  |  |  |  |  |  |  |
| Owuor 2019 | Low |  |  |  |  |  |  |  |
| Retnaningsih 2017 | Low |  |  |  |  |  |  |  |
| Van Melle | Low |  |  |  |  |  |  |  |
| Wang 2020 | Low |  |  |  |  |  |  |  |
| Westerling 2020 | Low |  |  |  |  |  |  |  |
